# RTA‐408 Enhances Radiosensitivity and Inhibited Tumor Progression via JNK Pathway in Glioblastoma

**DOI:** 10.1002/kjm2.70142

**Published:** 2025-11-28

**Authors:** Hung‐Pei Tsai, Hao Qin, Yoon Bin Chong, I‐Hsiang Chen, Shih‐Hsun Kuo, Tzu‐Ting Tseng, Ann‐Shung Lieu

**Affiliations:** ^1^ Division of Neurosurgery, Department of Surgery Kaohsiung Medical University Hospital Kaohsiung Taiwan; ^2^ Regenerative Medicine and Cell Therapy Research Center Kaohsiung Medical University Kaohsiung Taiwan; ^3^ Department of Neurosurgery Zaozhuang Municipal Hospital Zaozhuang China; ^4^ Graduate Institute of Medicine College of Medicine, Kaohsiung Medical University Kaohsiung Taiwan; ^5^ Department of Pathology Kaohsiung Medical University Hospital Kaohsiung Taiwan; ^6^ Department of Radiation Oncology Kaohsiung Medical University Hospital Kaohsiung Taiwan; ^7^ Department of Surgery, School of Medicine College of Medicine, Kaohsiung Medical University Kaohsiung Taiwan

**Keywords:** apoptosis, brain neoplasms, cell proliferation, neoplasm invasiveness, nuclear factor erythroid 2‐related factor

## Abstract

Glioblastoma (GBM) is an aggressive brain tumor with poor prognosis owing to its high invasiveness and resistance to therapy. RTA‐408, a synthetic triterpenoid and nuclear factor erythroid 2‐related factor 2 activator, exhibits anti‐inflammatory and anti‐cancer properties; however, its effects on GBM remain unclear. This study investigated the therapeutic potential of RTA‐408 in GBM, focusing on its role in the activation of the JNK pathway. GBM8401 and A172 cells were treated with RTA‐408, and cell viability, apoptosis, migration, and radiosensitivity were assessed. Western blot analysis was used to evaluate the epithelial‐mesenchymal transition markers, cyclin D1, and JNK signaling. Intracranial xenograft models were used to assess tumor growth suppression by RTA‐408 alone or in combination with radiotherapy. RTA‐408 significantly reduced cell viability, induced apoptosis, and inhibited the migration of GBM cells, correlating with the activation of the JNK pathway. JNK inhibition reversed these effects, confirming its role in RTA‐408‐mediated tumor suppression. RTA‐408 also enhanced radiosensitivity and reduced clonogenic survival. RTA‐408 suppressed GBM tumor growth in vivo, with the greatest effect observed in combination with radiotherapy. RTA‐408 exerts antitumour and radiosensitizing effects via activation of the JNK pathway and inhibits GBM progression. These findings highlight its potential as a novel therapeutic strategy for the treatment of GBM.

## 1 Introduction

Glioblastoma (GBM), also referred to as glioblastoma multiforme, is the most common and aggressive malignant primary brain tumor in adults, accounting for approximately 15% of all primary brain tumors and 51% of malignant gliomas [1, 2]. According to the definition of the World Health Organization for a grade IV astrocytoma, a GBM is characterized by significant intratumoural heterogeneity with high variability in genetic, epigenetic, and phenotypic profiles, contributing to its aggressive behavior, invasiveness, and resistance to treatment [3, 4, 5]. Despite current therapeutic strategies, including maximal surgical resection, radiotherapy (RT), and adjuvant chemotherapy with temozolomide, the prognosis for GBM remains poor, with a median survival of 14–21 months and a 5‐year survival rate of only 6.8% [6, 7, 8, 9]. Relapses frequently occur within 2 cm of the original surgical site in over 80% of cases, emphasizing the limitations of incomplete surgical resection and the development of multidrug resistance [10, 11]. These factors underscore the urgent need for novel therapeutic strategies to improve the outcomes of patients with GBM.

RTA‐408, also known as omaveloxolone, is a second‐generation synthetic oleanane triterpenoid and nuclear factor erythroid 2‐related factor 2 (Nrf2) activator that is currently being evaluated in clinical trials for the treatment of Friedreich's ataxia (FA), which has shown significant results in improving the neurological function of patients [12, 13]. This compound exerts antioxidant and anti‐inflammatory effects by activating Nrf2, inducing the transcription of cytoprotective genes, inhibiting the pro‐inflammatory nuclear factor‐kB (NF‐κB) pathway, and modulating inflammatory mediators, such as iNOS, 5‐LOX, and COX‐2 [14, 15, 16]. Preclinical studies have demonstrated that RTA‐408 and its precursor, CDDO‐Me, inhibit the growth of breast, lung, and prostate cancers [17, 18, 19, 20]. In addition, RTA‐408 has exhibited pharmacological effects in various diseases, including mitochondrial dysfunction, seizures, osteoporosis, and nonalcoholic steatohepatitis [21, 22, 23]. It has also been shown to protect mitochondrial membrane potential under oxidative stress, prevent oxidative cell death in fibroblasts of patients with FA, reverse neuropathic pain through mitochondrial biogenesis, and improve cardiovascular function in FA mouse models [24, 25, 26]. Furthermore, RTA‐408 significantly ameliorated cardiac oxidative stress, inflammatory responses, and cardiomyocyte apoptosis in a septic cardiomyopathy model, highlighting its cardioprotective properties [26]. Despite its broad pharmacological potential, there are no published studies on the effects of RTA‐408 on cancers, including GBM. Therefore, this study aimed to investigate the therapeutic potential of RTA‐408 in GBM.

## 2 Methods

### 2.1 Cell Lines and Culture Conditions

Two GBM cell lines, GBM8401 and A172, were used in this study. GBM8401 cells were obtained from the Bioresource Collection and Research Center (Taipei, Taiwan; 60,163), and A172 cells were obtained from the American Type Culture Collection (Manassas, VA, USA; CRL‐1620). GBM8401 cells were grown in Roswell Park Memorial Institute medium supplemented with 10% foetal bovine serum (FBS), and A172 cells were cultured in Dulbecco's modified Eagle's medium supplemented with 10% FBS. Both cell lines were maintained under standard conditions at 37°C in a humidified atmosphere containing 5% carbon dioxide.

### 2.2 Cell Viability Assay

Cell viability was assessed using the MTT assay method. GBM8401 and A172 cells were suspended in a culture medium containing 10% FBS, and plated in 24‐well plates at a density of approximately 30,000 cells/well for a total volume of 0.5 mL. The cells were incubated in 5% carbon dioxide at 37°C for 72 h. Following incubation, the cells were exposed to increasing concentrations of RTA‐408 (0, 50, 100, 200, 300, 400, 500, and 600 nM). Cell viability was determined by manually counting stained and unstained cells.

### 2.3 Migration Assay

The migration capability of GBM cells was evaluated using a wound‐healing assay (ibidi, Gräfelfing, Germany; 80209). A cell suspension of 1 × 10^6^ cells/mL (70 μL per well) was added to each well and allowed to adhere for 24 h. Non‐adherent cells were removed by washing twice with phosphate‐buffered saline (PBS). Cells were then treated with 50 nM RTA‐408, and the migration process was monitored over time. Images were captured to document cell movement into the wound area.

### 2.4 Colony Formation Assay

Colony formation was examined by plating GBM8401 and A172 cells in six‐well plates at different densities (100, 200, 400, 1000, and 10,000 cells per well). Cells were treated with RTA‐408 at concentrations of 50 nM, or with a JNK inhibitor, followed by irradiation at doses of 0, 1, 2, 4, and 8 Gy using a linear accelerator. After 10 days, the colonies were stained with 0.5% crystal violet and counted. Plating efficiency and surviving fraction were calculated relative to untreated controls.

### 2.5 Western Blot

To evaluate the protein expression levels associated with RTA‐408 treatment in glioblastoma cells, GBM8401 and A172 cells were lysed using 200 μL of lysis buffer on ice. The lysates were clarified by centrifugation, and the total protein concentration was quantified using a bicinchoninic acid (BCA) assay. Equal amounts (50 μg) of protein from each sample were resolved using sodium dodecyl sulfate‐polyacrylamide gel electrophoresis (SDS‐PAGE) at 50 V for 4 h and subsequently transferred onto polyvinylidene fluoride (PVDF) membranes. After blocking in 5% non‐fat milk at room temperature for 1 h, membranes were incubated overnight at 4°C with primary antibodies targeting JNK pathway activation markers and key regulators of epithelial‐mesenchymal transition (EMT), apoptosis, and DNA damage repair. The following primary antibodies were used: p‐p38 (#9211; 1:500; Cell Signaling; USA), p‐ERK (#9101; 1:500; Cell Signaling; USA), p‐JNK (#9251; 1:500; Cell Signaling; USA), β‐actin (A5441, 1:20,000, Sigma, USA), Cyclin D1 (60186–1‐lg, 1:500, Proteintech, Chicago, IL, USA), E‐cadherin (20874–1‐AP, 1:500, Proteintech, USA), and N‐cadherin (22018‐1‐AP, 1:500, Proteintech, USA). Following three washes with Tris‐buffered saline containing 0.1% Tween‐20 (TBST), the membranes were incubated with horseradish peroxidase (HRP)‐conjugated secondary antibodies—goat anti‐rabbit (AP132P, 1:5000, Millipore, Billerica, MA, USA) and goat anti‐mouse (AP124P, 1:5000, Millipore, USA)—for 90 min at room temperature. After a final series of washes, protein bands were visualized using enhanced chemiluminescence (ECL) reagent (Western Lightning, 205‐14621, Perkin Elmer, Waltham, MA, USA) and detected with a MiniChemi imaging and analysis system (Beijing Sage Creation, Beijing, China). The relative protein expression levels were quantified using ImageJ software, and β‐actin was used as an internal loading control. All experiments were performed in triplicate to ensure reproducibility.

### 2.6 Flow Cytometry

Apoptosis and cell cycle progression were analyzed by flow cytometry. For apoptosis analysis, 200,000 GBM8401 or A172 cells per well were cultured in six‐well plates for 72 h, followed by treatment with RTA‐408 at concentrations of 0, 50, 100, 200, and 400 nM, or with a JNK inhibitor. Cells were then labeled with annexin V and dead cell reagent (Merck Millipore, Warsaw, Poland) for 20 min at room temperature, and analyzed using a Muse Cell Analyzer (Merck Millipore). For cell cycle analysis, 10,000 cells per well were treated under similar conditions, fixed with cold 70% ethanol, and stored at −20°C. Prior to analysis, cells were rehydrated with PBS and stained according to the instructions of the manufacturer.

### 2.7 Animal Experiments

All animal procedures were conducted according to the protocols approved by the Institutional Animal Care and Use Committee of Kaohsiung Medical University (IACUC Approval No: 111223) and adhered to the relevant ethical guidelines. Immunodeficient NU/NU mice (LASCO Laboratory Animal Center, Taipei, Taiwan) were housed at 24°C under controlled light/dark cycles with free access to food and water. GBM8401 cells (100,000 cells in 5 μL) were stereotactically injected into the mouse striatum. One‐week post‐injection, the mice received intraperitoneal doses of 1 μM/kg RTA‐408. For radiosensitivity tests, the mice received 2 Gy radiation at 1 week after tumor cell implantation. Tumor progression was monitored using bioluminescence imaging with the Xenogen IVIS Spectrum system (IVIS Lumina LT 2D) at 7‐, 14‐, and 21‐days post‐injection.

### 2.8 Statistical Analyses

Data were processed using the SPSS software version 19.0 (Chicago, IL, USA). One‐way analysis of variance was used to assess differences among groups in the proliferation, migration, and invasion assays. Western blot results were analyzed using the Lane 1DTM software. Statistical significance was set at *p* < 0.05.

## 3 Results

### 3.1 Dose‐Dependent Effects of RTA‐408 on GBM Cell Viability and Apoptosis

**FIGURE 1:**
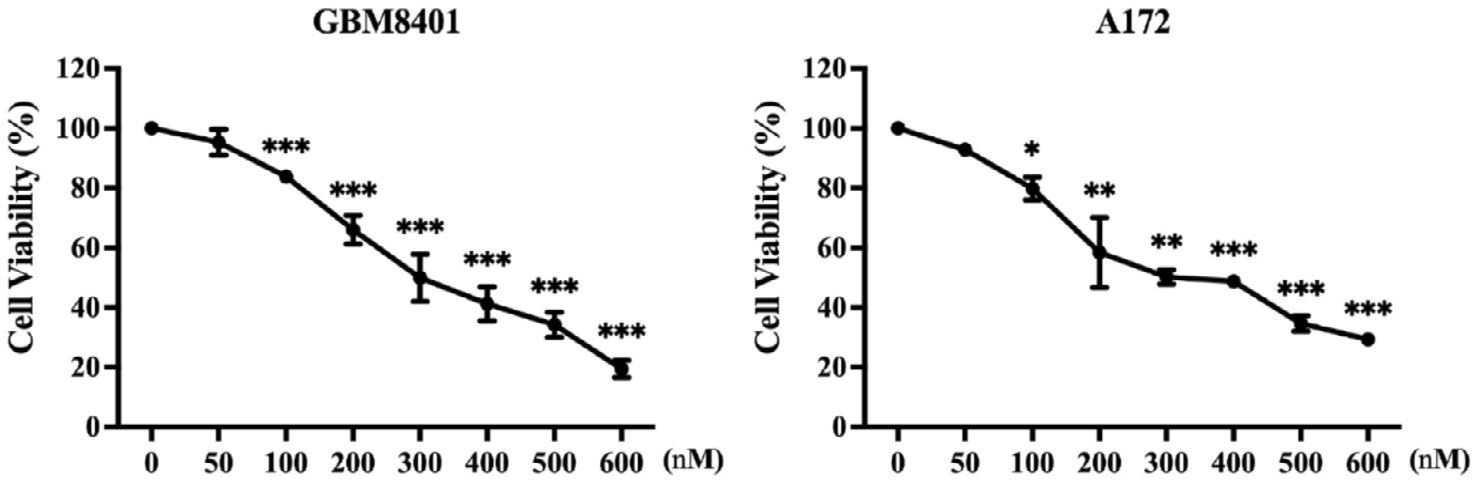
Effects of RTA‐408 on glioblastoma cell viability. GBM8401 and A172 cells were treated with increasing concentrations of RTA‐408 (0–600 nM) for 72 h and cell viability was assessed using the MTT assay. Data are presented as the mean ± standard error of the mean from at least three independent experiments. *p < 0.05, **p < 0.01, and ***p < 0.001 vs. control (0 nM).

To evaluate the cytotoxic effects of RTA‐408 on GBM cells, the viability of GBM8401 and A172 cells was assessed following treatment with increasing concentrations of RTA‐408 (0–600 nM). As shown in Figure 1, RTA‐408 significantly reduced the viability of GBM8401 and A172 cells in a dose‐dependent manner. In GBM8401 cells, a marked decrease in viability was observed at 100 nM, which further declined with increasing concentrations (*p* < 0.001). A172 cells exhibited a similar dose‐dependent reduction in viability, with significant effects observed at 100 nM (*p* < 0.05) and 200 nM (*p* < 0.01), and highly significant reductions at higher concentrations (*p* < 0.001). These results indicate that RTA‐408 effectively inhibited GBM cell growth in a concentration‐dependent manner.

**FIGURE 2:**
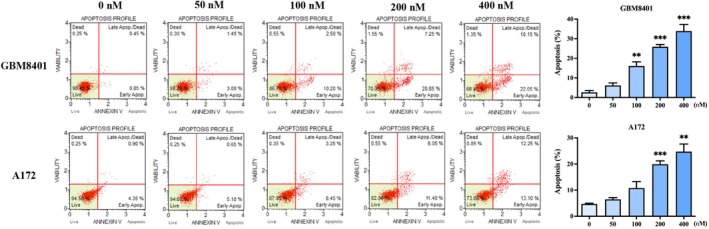
Effects of RTA‐408 on apoptosis in glioblastoma cells. GBM8401 and A172 cells were treated with increasing concentrations of RTA‐408 (0, 50, 100, 200, and 400 nM) for 72 h and apoptosis was assessed using annexin V/propidium iodide staining followed by flow cytometry. Representative scatter plots show the early and late apoptotic cell populations under each treatment condition. The bar graphs show the percentage of apoptotic cells (early and late apoptosis) in response to RTA‐408 treatment. Data are presented as the mean ± standard error of the mean from at least three independent experiments. **p < 0.01, ***p < 0.001 vs. control (0 nM).

To determine the pro‐apoptotic effects of RTA‐408 on GBM cells, apoptosis was analyzed in GBM8401 and A172 cells following treatment with increasing concentrations of RTA‐408 (0–400 nM) using annexin V/PI (propidium iodide) staining and flow cytometry (Figure 2). The results demonstrated a dose‐dependent increase in apoptotic cell populations in both cell lines. In GBM8401 cells, early and late apoptosis were significantly increased at 100 nM (*p* < 0.01), with more pronounced effects observed at 200 and 400 nM (*p* < 0.001). A172 cells exhibited a similar significant increase in apoptotic cell population starting at 100 nM (*p* < 0.05), more pronounced effects observed at 200 nM (*p* < 0.001) and 400 nM (*p* < 0.01). These findings indicate that RTA‐408 effectively induced apoptosis in GBM8401 and A172 cells in a dose‐dependent manner.

### 3.2 Dose‐Dependent Inhibition of GBM Cell Migration by RTA‐408

**FIGURE 3:**
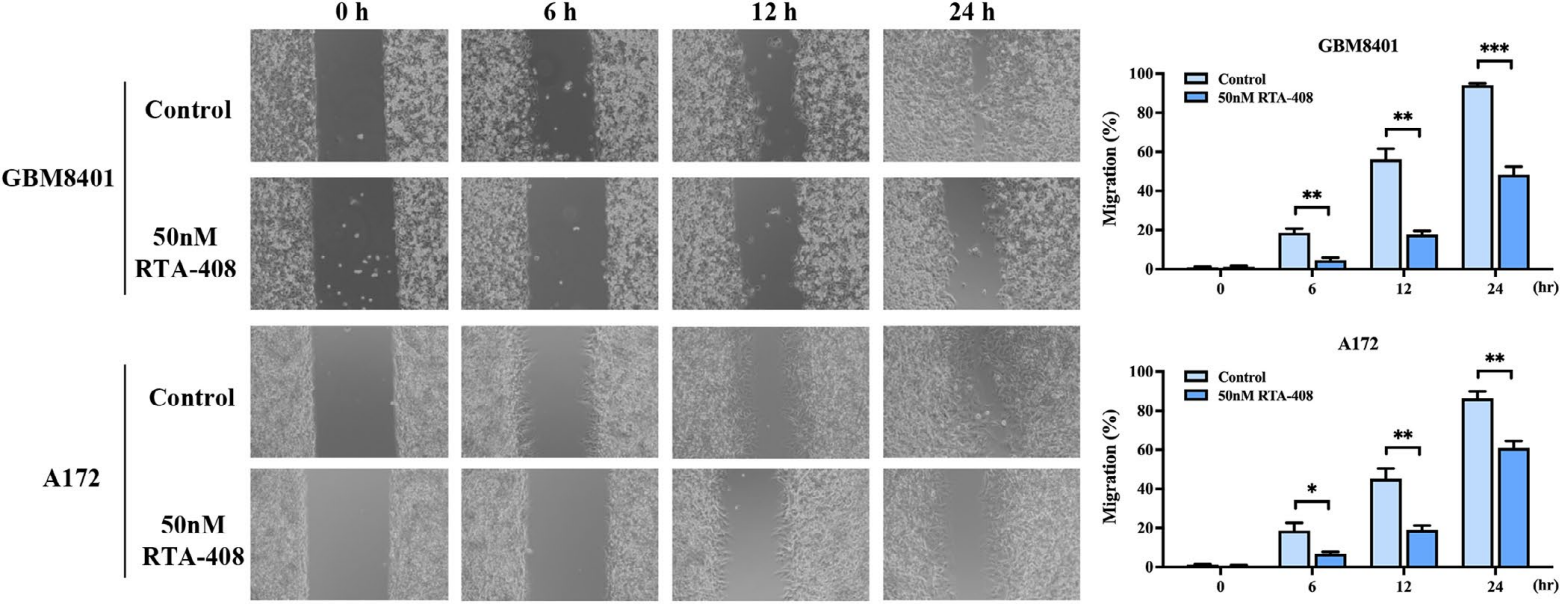
Effects of RTA‐408 on glioblastoma cell migration. GBM8401 and A172 cells were treated with 50 nM RTA‐408, and cell migration was assessed using a wound‐healing assay at 0, 6, 12, and 24 h. Representative images show wound closure at each time point in the control and RTA‐408‐treated cells. The bar graphs quantify the migration percentage over time. Data are presented as the mean ± standard error of the mean from at least three independent experiments. *p < 0.05, **p < 0.01, ***p < 0.001 vs. control (0 nM).

To evaluate the effect of RTA‐408 on GBM cell migration, a wound healing assay was performed using GBM8401 and A172 cells treated with 50 nM RTA‐408 over a 24‐h period. As shown in Figure 3, the untreated control cells exhibited time‐dependent closure of the wound area, indicating active migration. In contrast, the migration of RTA‐408‐treated cells significantly reduced at all time points. In GBM8401 cells, quantification of the percentage of wound closure revealed a significant decrease in migration at 6 h (*p* < 0.01), which became more pronounced at 12 h (*p* < 0.01) and 24 h (*p* < 0.001). In A172 cells, RTA‐408 treatment resulted in a significant reduction in migration compared to the control cells at 6 h (*p* < 0.05), which became more pronounced at 12 h (*p* < 0.01) and 24 h (*p* < 0.01). These results suggest that RTA‐408 effectively suppressed the migratory potential of GBM8401 and A172 cells, indicating its potential role in inhibiting GBM cell motility.

### 3.3 RTA‐408 Enhanced Radiosensitivity in GBM Cells in a Dose‐Dependent Manner

**FIGURE 4:**
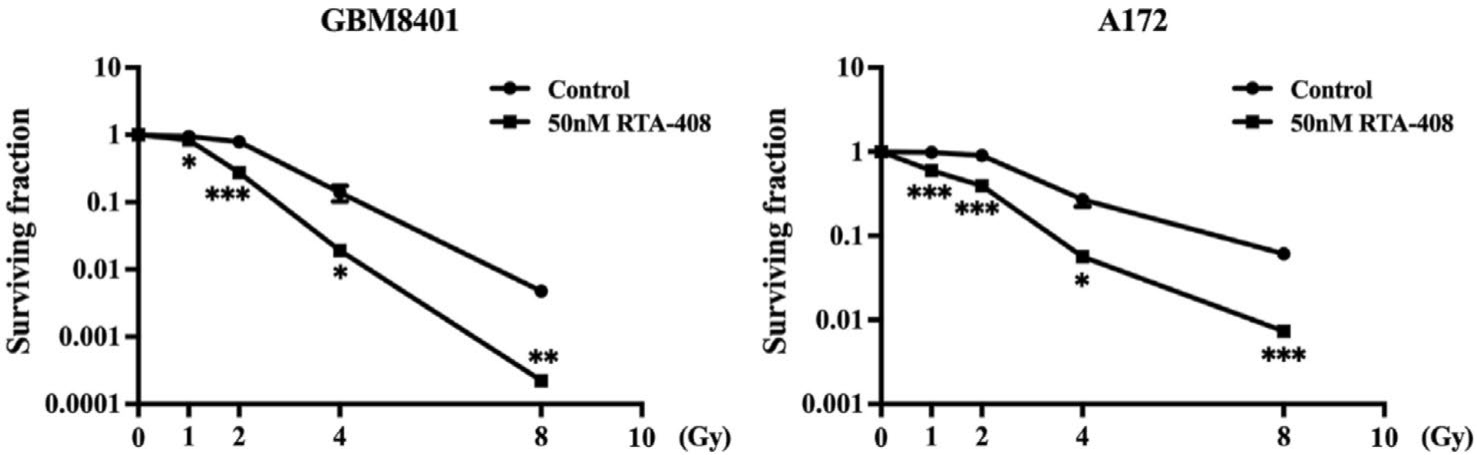
RTA‐408 enhanced radiosensitivity in glioblastoma cells. GBM8401 and A172 cells were treated with 50 nM RTA‐408 and exposed to increasing doses of radiation (0, 1, 2, 4, and 8 Gy). The clonogenic survival was assessed to determine the surviving fraction at each radiation dose. The results showed that RTA‐408 treatment significantly reduced cell survival in a dose‐dependent manner compared to the control group. Data are presented as the mean ± standard error of the mean from at least three independent experiments. *p < 0.05, **p < 0.01, ***p < 0.001 vs. control (0 nM).

To assess the effect of RTA‐408 on GBM radiosensitivity, a clonogenic survival assay was conducted using GBM8401 and A172 cells treated with 50 nM RTA‐408 followed by increasing doses of radiation (0, 1, 2, 4, and 8 Gy; Figure 4). The results demonstrated that RTA‐408 significantly enhanced the radiosensitivity of both cell lines. In GBM8401 cells, RTA‐408 treatment markedly reduced the surviving fraction at 1 (*p* < 0.05), 2 (*p* < 0.001), 4 (*p* < 0.05), and 8 Gy (*p* < 0.01), indicating a dose‐dependent radiosensitization effect. Similarly, in A172 cells, RTA‐408 significantly reduced survival at 2 (*p* < 0.001), 4 (*p* < 0.05), and 8 Gy (*p* < 0.001). These findings suggest that RTA‐408 enhances the susceptibility of GBM cells to radiation, thereby potentiating the effects of RT, which supports its potential as a radiosensitizing agent in GBM treatment.

### 3.4 Dose‐Dependent Suppression of Epithelial‐Mesenchymal Transition (EMT) and Cell Cycle Progression by RTA‐408 in GBM Cells

**FIGURE 5:**
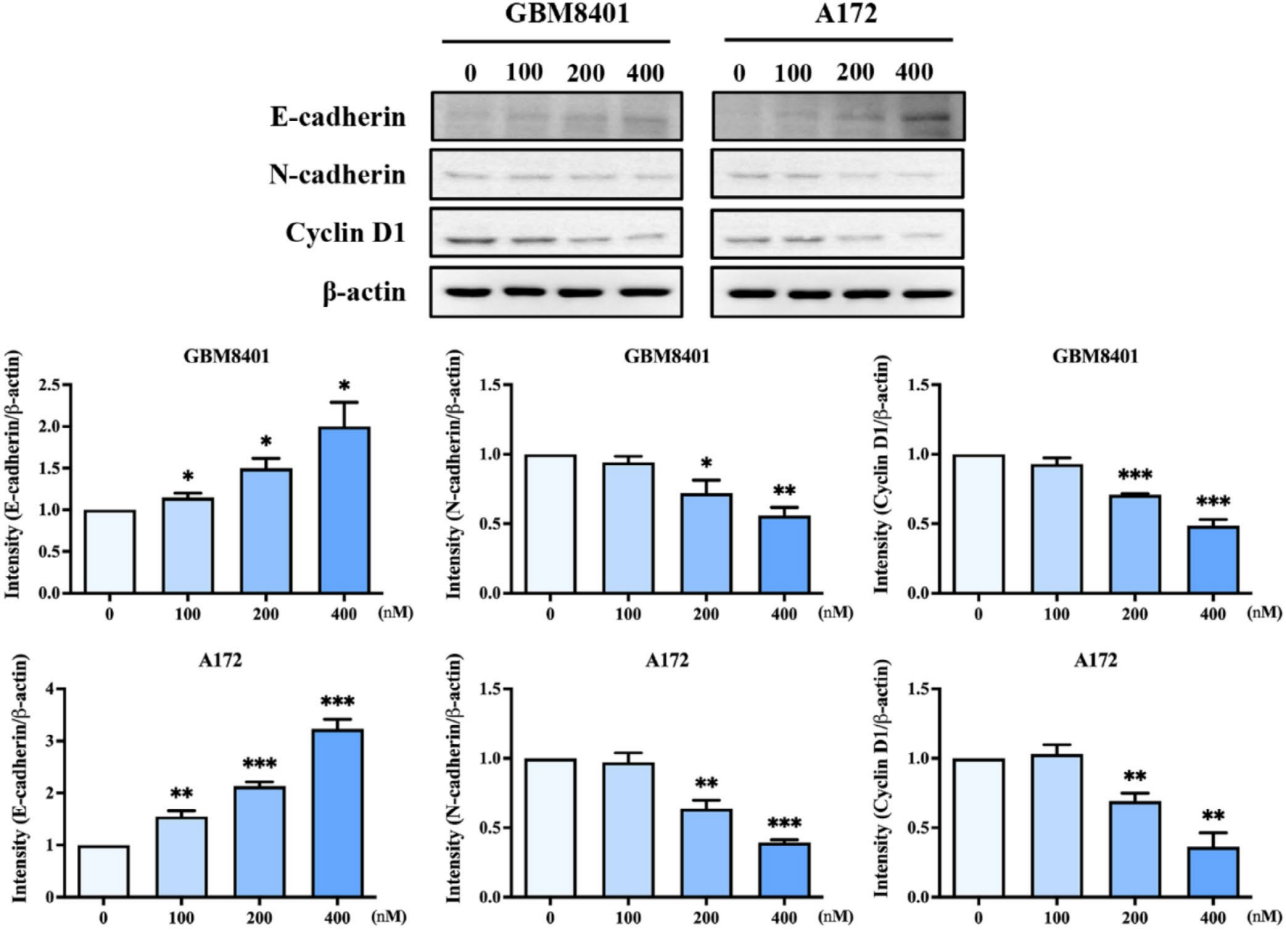
Effects of RTA‐408 on epithelial‐mesenchymal transition and cell cycle regulation in glioblastoma cells. GBM8401 and A172 cells were treated with increasing concentrations of RTA‐408 (0, 100, 200, and 400 nM) for 72 h and the expression levels of E‐cadherin, N‐cadherin, and cyclin D1 were analyzed by western blotting. β‐Actin was used as a loading control. Representative western blot images are shown (top), and the corresponding quantitative analysis of protein expression is displayed in the bar graphs (bottom). Data are presented as the mean ± standard error of the mean from at least three independent experiments. *p < 0.05, **p < 0.01, ***p < 0.001 vs. control (0 nM).

To investigate the effect of RTA‐408 on EMT and cell cycle regulation in GBM cells, western blotting was performed to assess the expression levels of E‐cadherin, N‐cadherin, and cyclin D1 in GBM8401 and A172 cells following treatment with increasing concentrations of RTA‐408 (0, 100, 200, and 400 nM; Figure 5). The results showed that RTA‐408 treatment significantly increased E‐cadherin expression in both GBM8401 and A172 cells in a dose‐dependent manner. In GBM8401 cells, E‐cadherin levels were significantly upregulated at 200 nM (*p* < 0.05) and 400 nM (*p* < 0.05). Similarly, in A172 cells, E‐cadherin levels were significantly upregulated at 100 nM (*p* < 0.01), 200 nM (*p* < 0.001), and 400 nM (*p* < 0.001). Conversely, the expression of N‐cadherin was significantly downregulated by RTA‐408 treatment, particularly at 200 nM (*p* < 0.05) and 400 nM (p < 0.01) in both cell lines, indicating EMT suppression. The expression of cyclin D1, a key regulator of cell‐cycle progression, was markedly reduced by RTA‐408 treatment in both GBM8401 and A172 cell lines at 200 nM (*p* < 0.001 and *p* < 0.01, respectively) and 400 nM (*p* < 0.001 and *p* < 0.01, respectively). These findings suggest that RTA‐408 suppresses GBM cell migration and proliferation by inhibiting EMT and downregulating cyclin D1, supporting its potential as an antitumour agent for GBM therapy.

### 3.5 RTA‐408 Exerts Anti‐Tumor Effects in GBM Through Activation of the JNK Pathway

**FIGURE 6:**
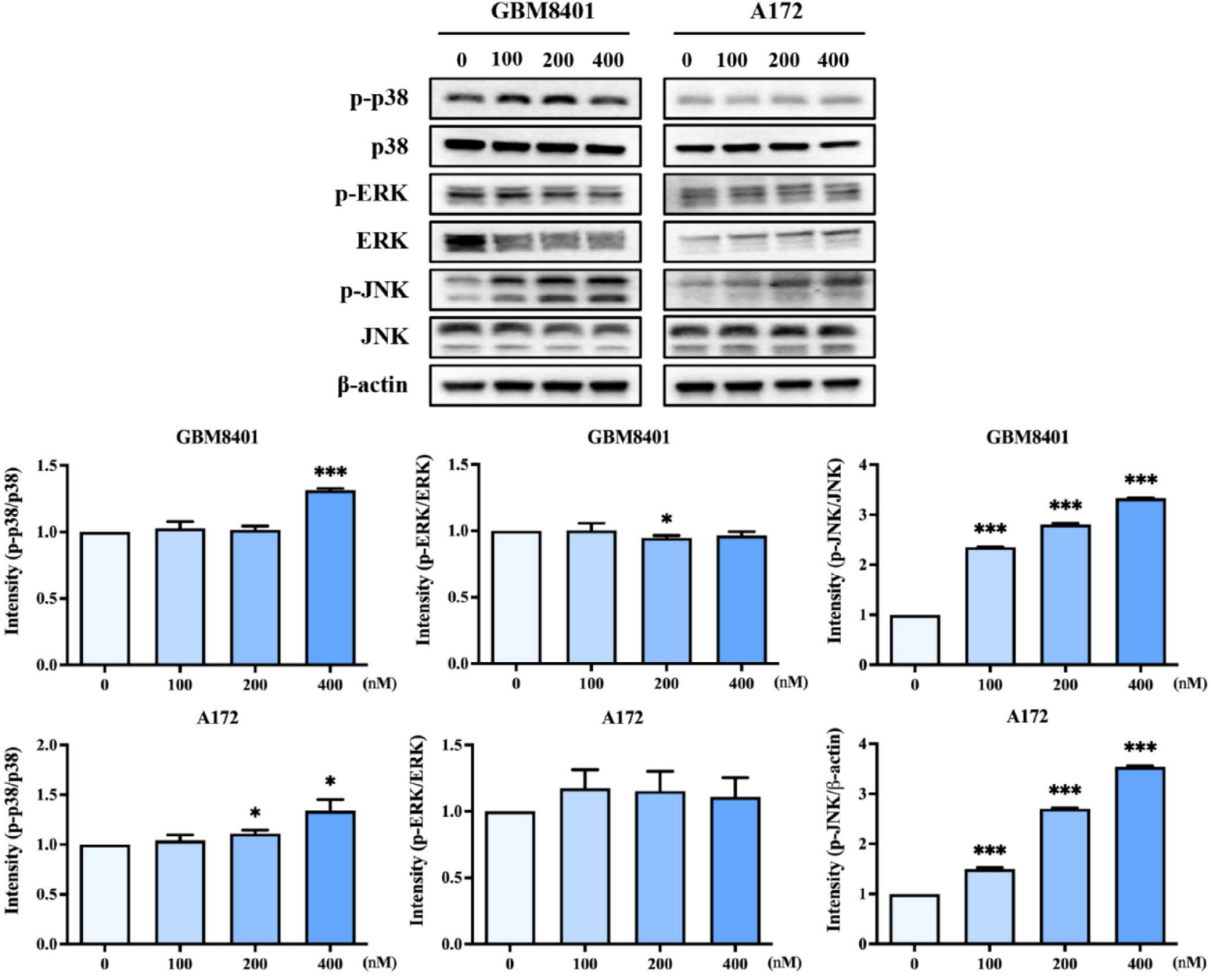
Effects of RTA‐408 on mitogen‐activated protein kinase signaling pathways in glioblastoma cells. GBM8401 and A172 cells were treated with increasing concentrations of RTA‐408 (0, 100, 200, and 400 nM) for 72 h and the ratio levels of p‐p38/p38, p‐ERK/ERK, and p‐JNK/JNK were analyzed by western blotting. β‐Actin was used as a loading control. Representative western blot images are shown (top), and the corresponding quantitative analysis of protein expression is displayed in the bar graphs (bottom). Data are presented as the mean ± standard error of the mean from at least three independent experiments. *p < 0.05, **p < 0.01, ***p < 0.001 vs. control (0 nM).

To explore the signaling mechanisms underlying the effects of RTA‐408 on GBM cells, western blotting was conducted to examine the ratio levels of p‐p38/p38, p‐ERK/ERK, and p‐JNK/JNK in GBM8401 and A172 cells following treatment with increasing concentrations of RTA‐408 (0, 100, 200, and 400 nM; Figure 6). The results demonstrated that RTA‐408 significantly increased p‐p38 expression in both cell lines. In GBM8401 cells, p‐p38 levels were significantly elevated at 400 nM (*p* < 0.05). In A172 cells, marked upregulation of p‐p38 was observed at 200 (*p* < 0.05) and 400 nM (*p* < 0.05). RTA‐408 treatment increased p‐ERK expression, particularly at high concentrations. In GBM8401 cells, p‐ERK/ERK significantly increased at 200 nM (*p* < 0.05). In A172 cells, p‐ERK/ERK ratio were no significantly increased at all dose. RTA‐408 significantly activated the JNK signaling pathway, as evidenced by a dose‐dependent increase in p‐JNK levels. In GBM8401 cells, p‐JNK/JNK ratio was significantly increased at 100 (*p* < 0.001), 200 (*p* < 0.001), and 400 nM (*p* < 0.001). Similarly, in A172 cells, p‐JNK levels were significantly increased at 100 nM (*p* < 0.001), 200 nM (p < 0.001) and further increased at 400 nM (*p* < 0.001). These findings suggest that RTA‐408 exerts its effects on GBM cells by activating the JNK, p38, and ERK signaling pathways. Notably, JNK activation exhibited a dose‐dependent increase in both GBM8401 and A172 cells, whereas p38 and ERK activation showed significant but less consistent trends at different concentrations. This indicates that JNK may play a central role in mediating the antitumour and pro‐apoptotic effects of RTA‐408 in GBM cells. Therefore, subsequent experiments were conducted using a JNK inhibitor to further investigate its role in RTA‐408‐mediated effects.

**FIGURE 7:**
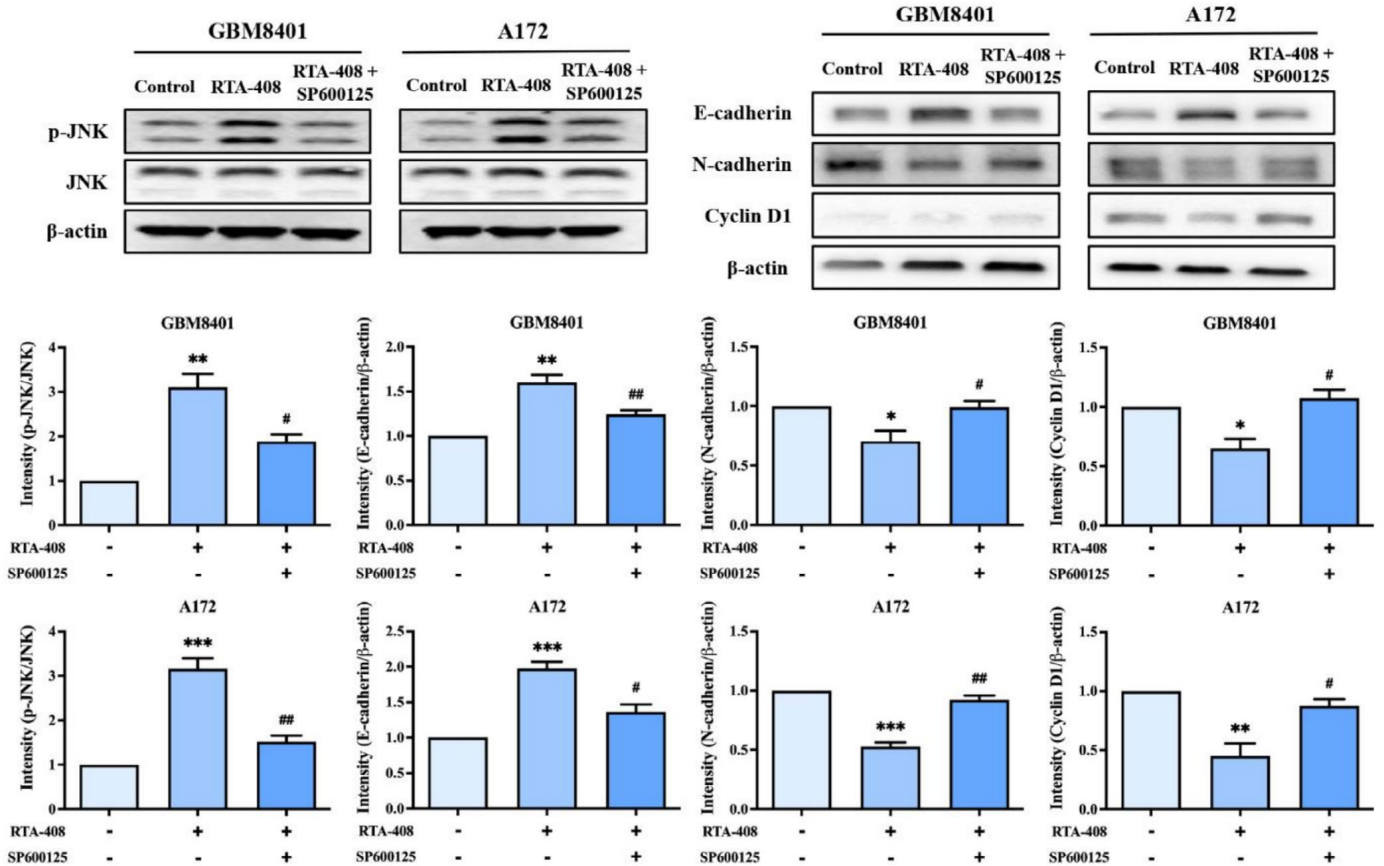
RTA‐408 activates JNK and suppresses EMT/Cyclin D1 via JNK signaling in GBM cells; effects are attenuated by the JNK inhibitor SP600125. GBM8401 and A172 cells were treated for 72 h with RTA‐408 (≈IC_50_; 400 nM) with or without the JNK inhibitor SP600125 (10 μM). Western blots for p‐JNK, total JNK, and β‐actin. Western blots for E‐cadherin, N‐cadherin, Cyclin D1, and β‐actin. Densitometric quantification: p‐JNK was normalized to total JNK; E‐cadherin, N‐cadherin, and Cyclin D1 were normalized to β‐actin. Data are presented as the mean ± standard error of the mean from at least three independent experiments. *p < 0.05, **p < 0.01, ***p < 0.001 vs. control (0 nM). (#*p* < 0.05, ##*p* < 0.01) vs. RTA‐408 alone.

To determine whether the effects of RTA‐408 on GBM cells were mediated through the JNK signaling pathway, western blot analysis was performed on GBM8401 and A172 cells treated with RTA‐408 alone or in combination with SP600125, a specific JNK inhibitor (Figure 7). The results showed that RTA‐408 significantly increased JNK phosphorylation (*p* < 0.01 in GBM8401 cells and *p* < 0.001 in A172 cells), confirming JNK pathway activation. However, the addition of SP600125 significantly reduced p‐JNK/JNK expression levels in both cell lines (*p* < 0.05 and *p* < 0.01, respectively), indicating effective inhibition of JNK signaling. In addition, RTA‐408 treatment upregulated E‐cadherin expression (*p* < 0.01 in GBM8401 cells and *p* < 0.001 in A172 cells) and downregulated N‐cadherin expression (*p* < 0.001 in GBM8401 cells and *p* < 0.01 in A172 cells), suggesting suppression of EMT. SP600125 reversed these effects, leading to reduced E‐cadherin and increased N‐cadherin expression (*p* < 0.05 and *p* < 0.01, respectively), further supporting JNK‐dependent EMT inhibition. Moreover, RTA‐408 significantly decreased cyclin D1 expression (*p* < 0.05 in GBM8401 cells and *p* < 0.01 in A172 cells), indicating a reduction in cell cycle progression. The addition of SP600125 restored cyclin D1 levels (*p* < 0.05), suggesting that RTA‐408 suppressed GBM proliferation through JNK activation. These findings confirm that RTA‐408 inhibits GBM cell proliferation and EMT progression via JNK pathway activation, and that inhibition of JNK signaling reverses these effects, further validating that JNK is a key mediator of RTA‐408‐induced tumor suppression.

### 3.6 JNK Pathway Mediates the Cytotoxic, Pro‐Apoptotic, Anti‐Migratory, and Radiosensitizing Effects of RTA‐408 in GBM Cells

**FIGURE 8:**
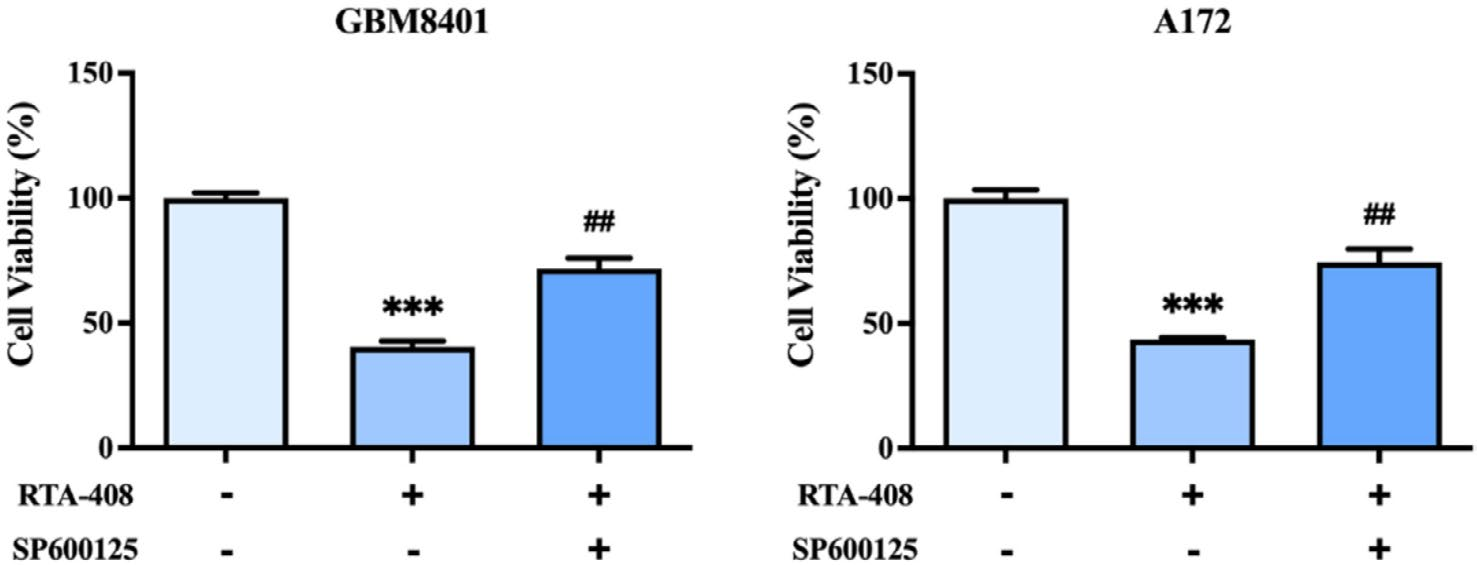
RTA‐408 reduces glioblastoma cell viability through the JNK signaling pathway. GBM8401 and A172 cells were treated with RTA‐408 (400 nM) alone or in combination with the JNK‐specific inhibitor SP600125 (10 μM) for 72 h. Cell viability was assessed using the MTT assay. The results showed that RTA‐408 significantly reduced cell viability compared to the control group (*p* < 0.001). Cotreatment with SP600125 significantly restored cell viability (^##^ *p* < 0.01), indicating that JNK inhibition mitigated the cytotoxic effects of RTA‐408. Data are presented as the mean ± standard error of the mean from at least three independent experiments. *p < 0.001 vs. control; ^##^ *p* < 0.01 vs. RTA‐408 treatment alone.

To determine whether the cytotoxic effects of RTA‐408 on GBM cells were mediated via the JNK pathway, cell viability was assessed in GBM8401 and A172 cells treated with RTA‐408 alone or in combination with SP600125 (Figure 8). The results showed that RTA‐408 treatment significantly reduced cell viability in both GBM8401 and A172 cells compared to the control group (*p* < 0.001), confirming its cytotoxic effect. Co‐treatment with SP600125 effectively reversed this reduction in cell viability, leading to a significant increase in cell survival compared to RTA‐408 alone (*p* < 0.01). These findings suggest that the anti‐proliferative effects of RTA‐408 on GBM cells are mediated through the JNK signaling pathway, as JNK inhibition mitigates RTA‐408‐induced cytotoxicity.

**FIGURE 9:**
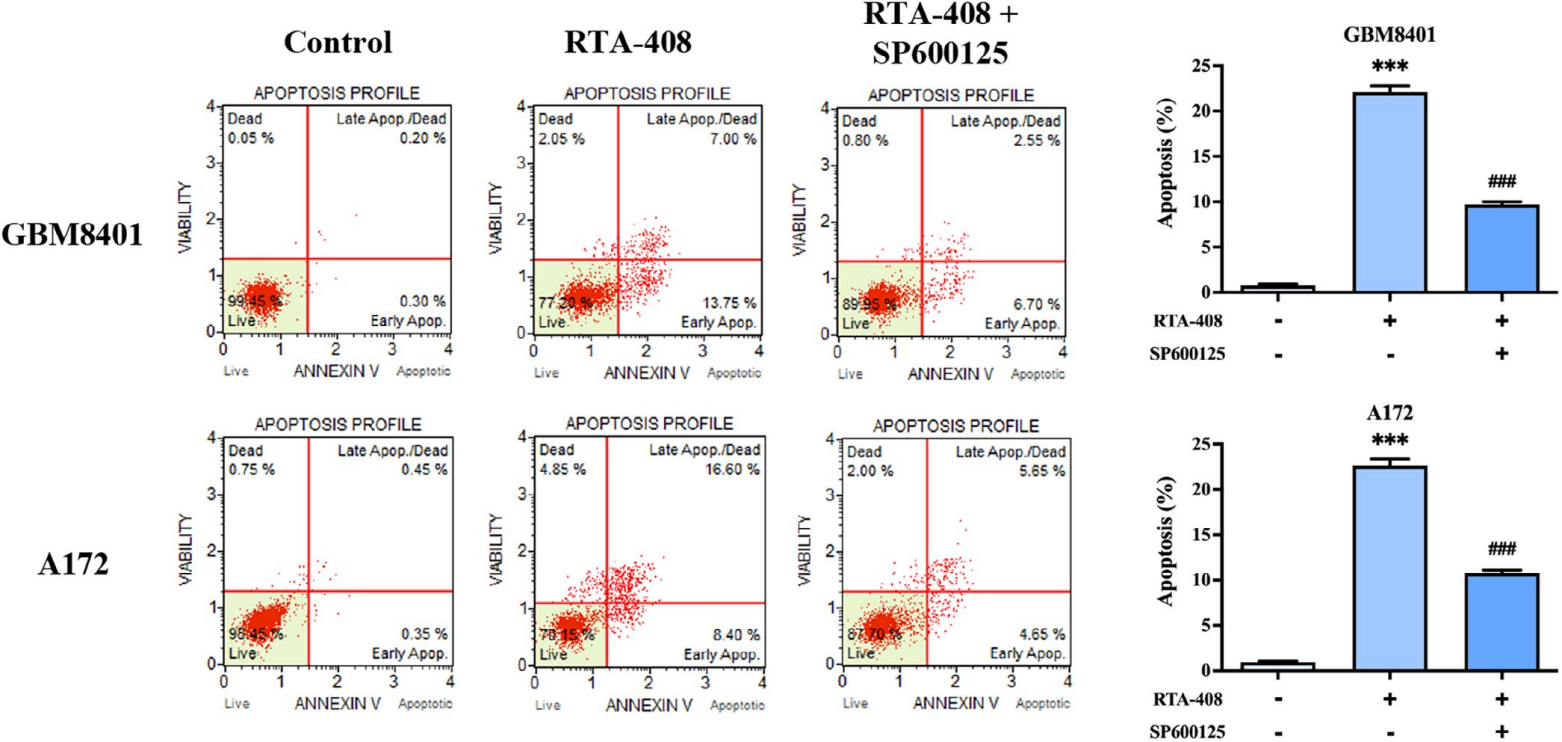
RTA‐408 induces apoptosis in glioblastoma cells via the JNK signaling pathway. GBM8401 and A172 cells were treated with RTA‐408 (400 nM) alone or in combination with the JNK‐specific inhibitor SP600125 (10 μM) for 72 h. Apoptosis was assessed using annexin V/propidium iodide staining, followed by flow cytometry. Representative scatter plots show the early and late apoptotic cell populations under each treatment condition. The bar graphs show the percentage of apoptotic cells (early and late apoptosis) in response to RTA‐408 treatment. RTA‐408 treatment significantly increased apoptosis compared to the control group (*p* < 0.001). Cotreatment with SP600125 significantly reduced apoptosis (^###^ *p* < 0.001), indicating that JNK inhibition attenuated RTA‐408‐induced apoptosis. Data are presented as the mean ± standard error of the mean from at least three independent experiments. *p < 0.001 vs. control; ^###^ *p* < 0.001 vs. RTA‐408 treatment alone.

To determine whether RTA‐408 induces apoptosis in GBM cells via the JNK signaling pathway, apoptosis was analyzed in GBM8401 and A172 cells treated with RTA‐408 alone or in combination with SP600125 using annexin V/PI staining and flow cytometry (Figure 9). The results showed that RTA‐408 treatment significantly increased apoptosis in both GBM8401 and A172 cells compared to the control group (*p* < 0.001), confirming its pro‐apoptotic effects. Co‐treatment with SP600125 markedly reduced the apoptotic cell population, leading to a significant decrease in apoptosis compared to treatment with RTA‐408 alone (*p* < 0.001). These findings indicate that RTA‐408 promotes apoptosis in GBM cells through the activation of the JNK signaling pathway, as inhibition of JNK signaling significantly attenuates RTA‐408‐induced apoptosis.

**FIGURE 10:**
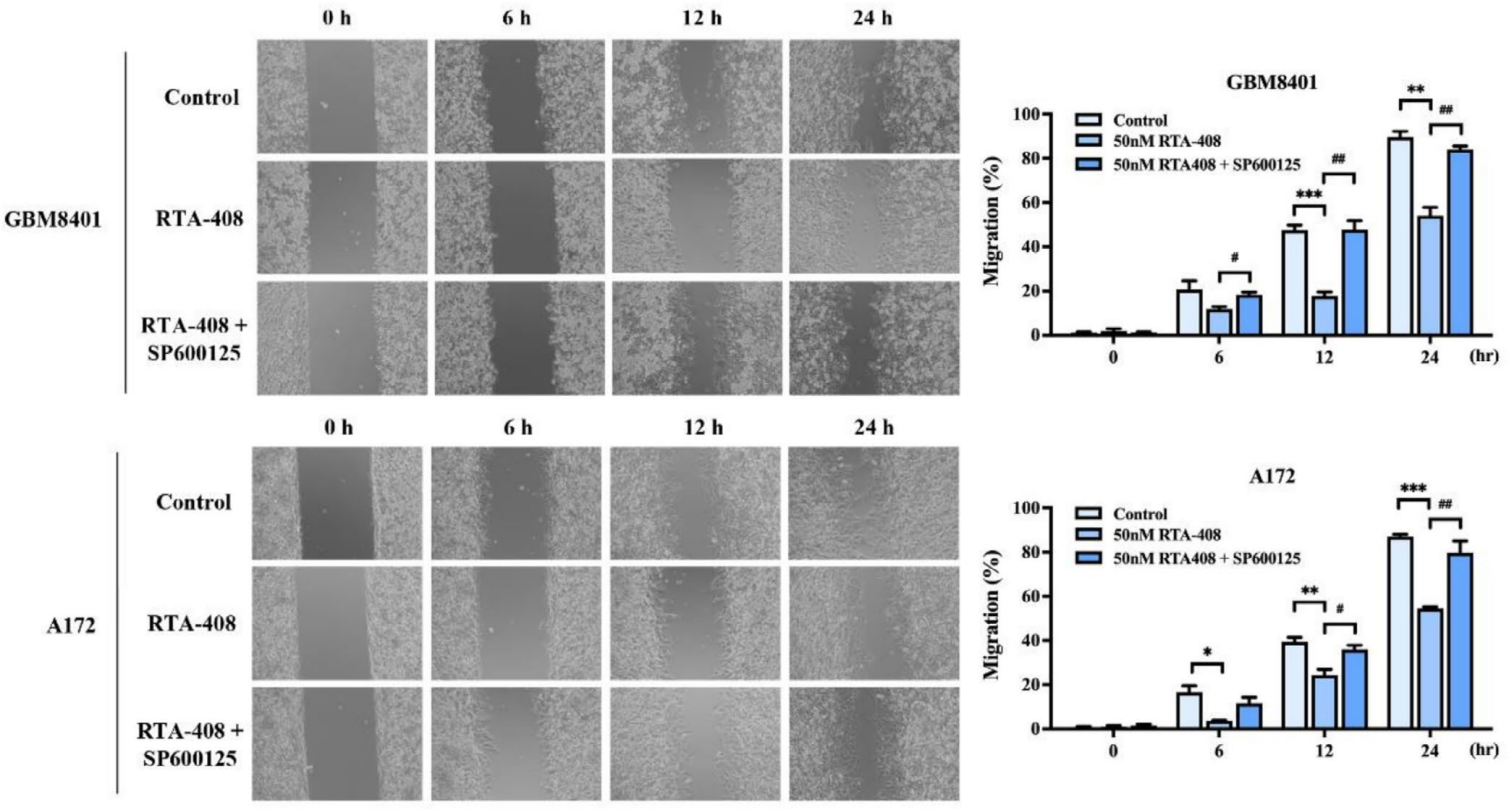
RTA‐408 inhibits glioblastoma cell migration through the JNK signaling pathway. GBM8401 and A172 cells were treated with RTA‐408 (50 nM) alone or in combination with the JNK‐specific inhibitor SP600125 (10 μM). Cell migration was assessed using a wound healing assay at 0, 6, 12, and 24 h. Representative images show wound closure under each treatment condition over time. Bar graphs represent the percentage of cell migration at each time point. RTA‐408 treatment significantly inhibited cell migration compared to the control group (*p* < 0.001), whereas co‐treatment with SP600125 partially restored migration at 6 h (^#^ *p* < 0.05), 12 h (^##^ *p* < 0.01), and 24 h (^##^ *p* < 0.01), indicating that JNK inhibition attenuated the anti‐migratory effects of RTA‐408. Data are presented as the mean ± standard error of the mean from at least three independent experiments. *p < 0.05, **p < 0.01, ***p < 0.001 vs. control; ^#^ *p* < 0.05, ^##^p < 0.01 vs. RTA‐408 treatment alone.

To evaluate whether RTA‐408 suppressed GBM cell migration via the JNK signaling pathway, a wound healing assay was performed on GBM8401 and A172 cells treated with RTA‐408 alone or in combination with SP600125 (Figure 10). The results showed that RTA‐408 significantly reduced the migration of both GBM8401 and A172 cells at all time points compared to the control group (*p* < 0.001), confirming its inhibitory effect on cell motility. Co‐treatment with SP600125 partially restored migration ability, leading to a significant increase in wound closure at 6 h (*p* < 0.05), 12 h (*p* < 0.01), and 24 h (*p* < 0.01) compared with RTA‐408 treatment alone. These findings suggest that RTA‐408 inhibits GBM cell migration by activating the JNK pathway, as JNK inhibition partially reverses its suppressive effect on cell motility.

**FIGURE 11:**
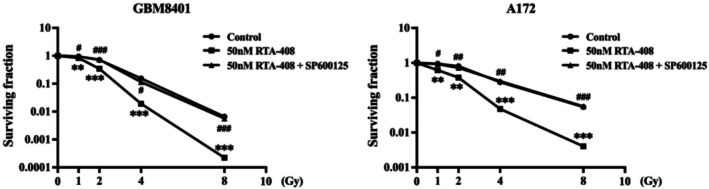
RTA‐408 enhances radiosensitivity in glioblastomacells through the JNK signaling pathway. GBM8401 and A172 cells were treated with RTA‐408 (50 nM) alone or in combination with the JNK‐specific inhibitor SP600125 (10 μM), followed by increasing doses of radiation (0, 1, 2, 4, and 8 Gy). The clonogenic survival was assessed to determine the surviving fraction at each radiation dose. RTA‐408 treatment significantly reduced the surviving fraction in a dose‐dependent manner compared to the control group (*p* < 0.001), indicating enhanced radiosensitivity. Co‐treatment with SP600125 significantly increased survival compared to RTA‐408 alone at 1 (^#^ *p* < 0.05), 2 (^##^ *p* < 0.01), 4 (^###^ *p* < 0.001), and 8 (^####^ *p* < 0.0001), suggesting that JNK inhibition attenuated the radiosensitizing effects of RTA‐408. Data are presented as the mean ± standard error of the mean from at least three independent experiments. **p < 0.01, *p < 0.001 vs. control; ^#^ *p* < 0.05, ^##^ *p* < 0.01, ^###^ *p* < 0.001, ^####^ *p* < 0.0001 vs. RTA‐408 treatment alone.

To determine whether RTA‐408 enhanced GBM radiosensitivity through the JNK signaling pathway, a clonogenic survival assay was performed on GBM8401 and A172 cells treated with RTA‐408 alone or in combination with SP600125, followed by increasing doses of radiation (0, 1, 2, 4, and 8 Gy; Figure 11). The results demonstrated that RTA‐408 significantly enhanced the radiosensitivity of both GBM cell lines, as shown by a marked reduction in the surviving fraction compared to the control group at all radiation doses (*p* < 0.001). Co‐treatment with SP600125 significantly increased survival compared with RTA‐408 alone at 1 (*p* < 0.05), 2 (p < 0.01), 4 (*p* < 0.001), and 8 Gy (*p* < 0.0001), indicating that JNK inhibition partially reversed the radiosensitizing effect of RTA‐408. These findings suggest that RTA‐408 enhances GBM radiosensitivity through activation of the JNK pathway, as inhibition of JNK signaling mitigates its radiosensitizing effects.

### 3.7 RTA‐408 Suppresses GBM Tumor Growth In Vivo and Enhances the Therapeutic Efficacy of RT

**FIGURE 12:**
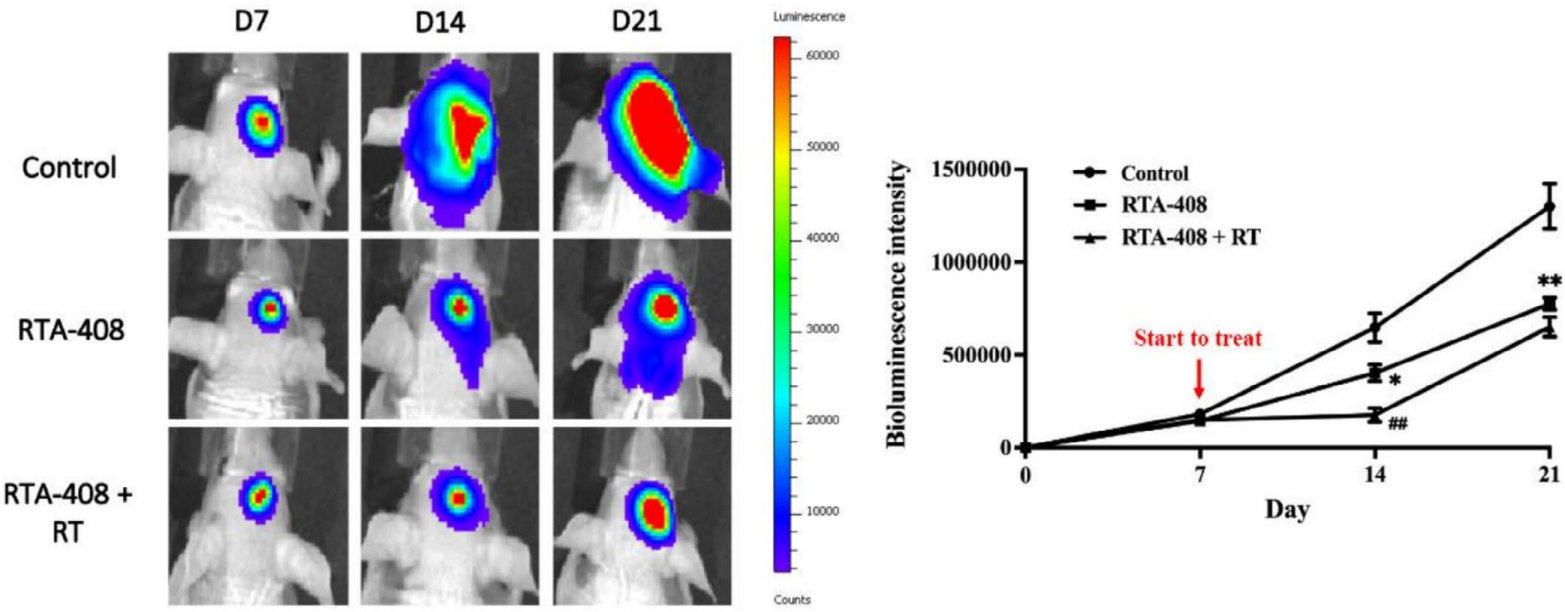
RTA‐408 suppresses GBM tumor growth in vivo and enhances the therapeutic effect of RT. Bioluminescence imaging was used to monitor tumor progression in intracranial GBM‐bearing mice treated with RTA‐408 alone or in combination with RT. Treatment was initiated on day 7 post‐implantation, as indicated by the red arrows. Representative bioluminescence images of tumor‐bearing mice are shown on days 7, 14, and 21, with color intensity representing tumor burden. The right panel quantifies bioluminescence intensity over time in each treatment group. Tumor growth was significantly inhibited in the RTA‐408‐treated group compared to the control group (*p < 0.05, *p < 0.01). The combination of RTA‐408 and RT further suppressed tumor progression, showing a significant reduction in bioluminescence intensity compared with RTA‐408 alone (##*p* < 0.01). Data are presented as mean ± standard error of the mean. *p < 0.05, *p < 0.01 vs. control; ##*p* < 0.01 vs. RTA‐408 treatment alone. GBM, glioblastoma; RT, radiotherapy.

To evaluate the effects of RTA‐408 alone and in combination with RT on GBM tumor growth in vivo, bioluminescence imaging was performed on intracranial tumor‐bearing mice on days 7, 14, and 21 after treatment initiation (Figure 12). Bioluminescence images demonstrated a significant increase in tumor burden in the control group over time, whereas RTA‐408 treatment alone effectively suppressed tumor growth. The combination of RTA‐408 and RT resulted in the most pronounced reduction in tumor progression. Quantification of bioluminescence intensity further confirmed these findings, showing a significantly lower tumor burden in the RTA‐408 group than in the control group on days 14 (*p* < 0.05) and 21 (*p* < 0.01). Furthermore, the combination treatment group exhibited even greater tumor suppression, with a significantly lower bioluminescence intensity than the RTA‐408 alone group on day 14 (*p* < 0.01). These findings suggest that RTA‐408 effectively suppresses GBM tumor growth in vivo, and its combination with RT further enhances its therapeutic efficacy.

## 4 Discussion

CDDO, a synthetic derivative of oleanolic acid, is a novel triterpenoid known for its anti‐inflammatory and antioxidant properties through modulation of Nrf2 and NF‐κB signaling [27]. To enhance its therapeutic potential, new derivatives, such as CDDO‐Me, CDDO‐ethyl amide, and CDDO‐Im have been developed [28, 29, 30, 31]. These derivatives have demonstrated anticancer efficacy in various models: CDDO‐Me suppresses non‐small cell lung cancer by inhibiting EGFR mutations [28], CDDO‐Im induces apoptosis in myeloma via unfolded protein response activation [29], and CDDO‐EA reduces inflammation in colorectal cancer [31]. RTA‐408 is the most recent derivative, and its pharmacological benefits extend to various diseases, including neurodegenerative disorders, cardiovascular diseases, and metabolic syndromes. RTA‐408 has demonstrated therapeutic potential in conditions such as FA, nonalcoholic steatohepatitis, diabetic cardiomyopathy, and osteoporotic bone loss [32].

The mechanism of action of RTA‐408 revolves around its ability to upregulate Nrf2, a master regulator of antioxidant defense, while concurrently suppressing NF‐κB‐driven inflammation. This dual function of RTA‐408 makes it an effective agent against chronic inflammatory diseases and oxidative stress‐induced cellular damage. In the context of neuropathic pain, RTA‐408 has been shown to alleviate nociceptive hypersensitivity by regulating p‐NF‐κB/TSLP/STAT5 signaling, reducing neuronal apoptosis and glial cell activation [17]. Furthermore, its role in cardiovascular protection has been confirmed in diabetic cardiomyopathy models, wherein it ameliorates oxidative stress, suppresses mitochondrial dysfunction, and improves cardiac function [32]. RTA‐408 has also garnered attention in cancer therapy because of its ability to inhibit tumor cell proliferation and enhance apoptosis. As an antioxidant inflammatory modulator, RTA‐408 directly inhibits NF‐κB signaling and reduces cyclin D1 expression, leading to cell cycle arrest and apoptosis in multiple cancer models. Preclinical studies have indicated that RTA‐408 significantly suppresses tumor growth by modulating the oxidative stress and inflammatory pathways [33]. In addition, it enhances the radiosensitivity of cancer cells, making it a promising adjunct to RT [17].

In this study, we investigated the therapeutic potential of RTA‐408 against GBM, and our findings demonstrate that RTA‐408 effectively reduced GBM cell viability and induced apoptosis in a dose‐dependent manner. RTA‐408 enhances the phosphorylation of JNK, a critical mediator of stress‐induced apoptosis, while concurrently suppressing EMT. Inhibition of EMT, as evidenced by increased E‐cadherin and decreased N‐cadherin expression, suggests that RTA‐408 impairs GBM cell migration and invasiveness. Furthermore, our study highlights the radiosensitizing effect of RTA‐408, as its combination with RT significantly reduced GBM clonogenic survival compared with radiation alone.

The JNK pathway is a crucial component of the mitogen‐activated protein kinase (MAPK) family that responds to various extracellular stimuli including oxidative stress, inflammatory cytokines, and DNA damage. JNK activation occurs through a cascade of phosphorylation events, leading to the activation of transcription factors, such as activator protein‐1 (AP‐1), c‐Jun, and p53. These transcriptional regulators modulate cell survival, apoptosis, differentiation, and inflammatory responses [34, 35]. JNK has been identified as a double‐edged sword in cancer, with both tumor‐suppressive and tumor‐promoting roles. In some malignancies, JNK activation leads to pro‐apoptotic signaling through the induction of the Bcl‐2‐associated death promoter and Bcl‐2‐like protein 11, and the activation of caspase‐dependent apoptotic pathways. However, in other cancers, persistent JNK activation contributes to tumor progression by promoting EMT, metastasis, and therapy resistance [34]; therefore, targeting JNK signaling represents a complex but promising therapeutic approach for different cancer types [33].

GBM is highly resistant to conventional treatments, such as RT and chemotherapy [36]. JNK activation in GBM has been associated with diverse effects depending on the tumor microenvironment and specific genetic alterations. Studies have suggested that JNK can induce apoptosis in GBM cells under certain conditions; however, paradoxically, it may also contribute to therapy resistance and tumor survival by promoting inflammatory signaling and tumor cell adaptation to hypoxic stress [37, 38]. Several studies have linked JNK activation in GBM to the regulation of GBM stem‐like cells, which are responsible for tumor recurrence and resistance to therapy [38]. The role of JNK in the regulation of EMT, tumor invasion, and proliferation underscores the importance of understanding its precise molecular functions in GBM pathophysiology [39].

Beyond its well‐characterized role in neuroprotection and mitochondrial function, emerging evidence suggests that RTA‐408 modulates tumor cell survival by influencing stress‐related MAPK signaling pathways, including the JNK pathway. In this study, we explored the pro‐apoptotic effects of RTA‐408 in GBM through JNK activation. Our findings demonstrated that RTA‐408 treatment led to a significant increase in JNK phosphorylation, which correlated with enhanced apoptosis in GBM8401 and A172 cells. This suggests that RTA‐408 exerts its antitumour effects in GBM, at least in part, through JNK pathway activation. Activation of the JNK pathway by RTA‐408 led to apoptosis while simultaneously suppressing EMT markers, such as N‐cadherin. Suppression of cyclin D1 further confirmed that RTA‐408 disrupted GBM cell cycle progression, thereby reducing tumor cell proliferation. The inhibition of JNK using SP600125 effectively reversed the apoptotic and antiproliferative effects of RTA‐408, demonstrating that JNK activation is a critical mediator of RTA‐408‐induced cytotoxicity in GBM cells.

RT remains the cornerstone of GBM treatment; however, its effectiveness is limited by intrinsic and acquired radioresistance. JNK activation has been implicated in the modulation of radiosensitivity. Our results indicated that RTA‐408 significantly enhanced GBM cell radiosensitivity by inducing apoptosis through JNK signaling. Cells treated with RTA‐408 in combination with RT exhibited lower clonogenic survival rates than those treated with RT alone. This suggests that RTA‐408 potentiates the effects of RT by amplifying JNK‐mediated apoptotic signaling. Interestingly, inhibition of JNK using SP600125 rescued GBM cells from RTA‐408‐induced radiosensitization, further validating that JNK activation plays a crucial role in enhancing GBM radiosensitivity. These findings support the potential of RTA‐408 as a radiosensitizer in GBM therapy.

In conclusion, this study demonstrated that RTA‐408 exhibited potent antitumour effects in GBM by reducing cell viability, inducing apoptosis, inhibiting migration, and enhancing radiosensitivity in a dose‐dependent manner. RTA‐408 exerts these effects through the activation of the JNK signaling pathway, as JNK inhibition with SP600125 reverses its cytotoxic, pro‐apoptotic, and anti‐migratory activities. Furthermore, RTA‐408 downregulated EMT markers and cyclin D1, suggesting its role in suppressing tumor cell proliferation and invasion. In vivo, RTA‐408 effectively inhibited tumor growth in the intracranial GBM mouse model. The combination of RTA‐408 and RT provided enhanced therapeutic efficacy. These findings highlight the potential of RTA‐408 as a novel therapeutic agent for GBM, particularly in combination with RT. Targeting the JNK pathway with RTA‐408 may be a promising strategy for improving GBM treatment outcomes and overcoming resistance to therapy.

## Conflicts of Interest

The authors declare no conflicts of interest.

## Data Availability

Data sharing not applicable to this article as no datasets were generated or analyzed during the current study.

## References

[1] C. C. da Hora , M. W. Schweiger , T. Wurdinger , and B. A. Tannous , “Patient‐Derived Glioma Models: From Patients to Dish to Animals,” Cells 8, no. 10 (2019): 1177. 31574953 10.3390/cells8101177 PMC6829406

[2] Q. T. Ostrom , N. Patil , G. Cioffi , K. Waite , C. Kruchko , and J. S. Barnholtz‐Sloan , “CBTRUS Statistical Report: Primary Brain and Other Central Nervous System Tumors Diagnosed in the United States in 2013–2017,” Neuro‐Oncology 22, no. 12 Suppl 2 (2020): iv1–iv96. 33123732 10.1093/neuonc/noaa200 PMC7596247

[3] D. N. Louis , A. Perry , P. Wesseling , et al., “The 2021 WHO Classification of Tumors of the Central Nervous System: A Summary,” Neuro‐Oncology 23, no. 8 (2021): 1231–1251. 34185076 10.1093/neuonc/noab106 PMC8328013

[4] N. Rabah , F. E. Ait Mohand , and N. Kravchenko‐Balasha , “Understanding Glioblastoma Signaling, Heterogeneity, Invasiveness, and Drug Delivery Barriers,” International Journal of Molecular Sciences 24, no. 18 (2023): 14256. 37762559 10.3390/ijms241814256 PMC10532387

[5] E. Le Rhun , M. Preusser , P. Roth , et al., “Molecular Targeted Therapy of Glioblastoma,” Cancer Treatment Reviews 80 (2019): 101896. 31541850 10.1016/j.ctrv.2019.101896

[6] Q. T. Ostrom , G. Cioffi , H. Gittleman , et al., “CBTRUS Statistical Report: Primary Brain and Other Central Nervous System Tumors Diagnosed in the United States in 2012–2016,” Neuro‐Oncology 21, no. Suppl 5 (2019): v1–v100. 31675094 10.1093/neuonc/noz150 PMC6823730

[7] Q. T. Ostrom , D. J. Cote , M. Ascha , C. Kruchko , and J. S. Barnholtz‐Sloan , “Adult Glioma Incidence and Survival by Race or Ethnicity in the United States From 2000 to 2014,” JAMA Oncology 4, no. 9 (2018): 1254–1262. 29931168 10.1001/jamaoncol.2018.1789 PMC6143018

[8] R. Stupp , M. E. Hegi , W. P. Mason , et al., “Effects of Radiotherapy With Concomitant and Adjuvant Temozolomide Versus Radiotherapy Alone on Survival in Glioblastoma in a Randomised Phase III Study: 5‐Year Analysis of the EORTC‐NCIC Trial,” Lancet Oncology 10, no. 5 (2009): 459–466. 19269895 10.1016/S1470-2045(09)70025-7

[9] R. Lakomy , T. Kazda , I. Selingerova , et al., “Real‐World Evidence in Glioblastoma: Stupp's Regimen After a Decade,” Frontiers in Oncology 10 (2020): 840. 32719739 10.3389/fonc.2020.00840 PMC7348058

[10] M. Waqar , F. Roncaroli , E. J. Lehrer , et al., “Rapid Early Progression (REP) of Glioblastoma Is an Independent Negative Prognostic Factor: Results From a Systematic Review and Meta‐Analysis,” Neuro‐Oncology Advances 4, no. 1 (2022): vdac075. 35769410 10.1093/noajnl/vdac075 PMC9234755

[11] M. Jezierzanski , N. Nafalska , M. Stopyra , et al., “Temozolomide (TMZ) in the Treatment of Glioblastoma Multiforme‐A Literature Review and Clinical Outcomes,” Current Oncology 31, no. 7 (2024): 3994–4002. 39057168 10.3390/curroncol31070296 PMC11275351

[12] D. R. Lynch and J. Johnson , “Omaveloxolone: Potential New Agent for Friedreich Ataxia,” Neurodegenerative Disease Management 11, no. 2 (2021): 91–98. 33430645 10.2217/nmt-2020-0057

[13] D. R. Lynch , M. P. Chin , M. B. Delatycki , et al., “Safety and Efficacy of Omaveloxolone in Friedreich Ataxia (MOXIe Study),” Annals of Neurology 89, no. 2 (2021): 212–225. 33068037 10.1002/ana.25934 PMC7894504

[14] J. M. Patlolla and C. V. Rao , “Triterpenoids for Cancer Prevention and Treatment: Current Status and Future Prospects,” Current Pharmaceutical Biotechnology 13, no. 1 (2012): 147–155. 21466427 10.2174/138920112798868719

[15] R. Ahmad , D. Raina , C. Meyer , S. Kharbanda , and D. Kufe , “Triterpenoid CDDO‐Me Blocks the NF‐kappaB Pathway by Direct Inhibition of IKKbeta on Cys‐179,” Journal of Biological Chemistry 281, no. 47 (2006): 35764–35769. 16998237 10.1074/jbc.M607160200

[16] K. Liby , T. Hock , M. M. Yore , et al., “The Synthetic Triterpenoids, CDDO and CDDO‐Imidazolide, Are Potent Inducers of Heme Oxygenase‐1 and Nrf2/ARE Signaling,” Cancer Research 65, no. 11 (2005): 4789–4798. 15930299 10.1158/0008-5472.CAN-04-4539

[17] V. Alexeev , E. Lash , A. Aguillard , et al., “Radiation Protection of the Gastrointestinal Tract and Growth Inhibition of Prostate Cancer Xenografts by a Single Compound,” Molecular Cancer Therapeutics 13, no. 12 (2014): 2968–2977. 25398830 10.1158/1535-7163.MCT-14-0354 PMC4258451

[18] M. S. Ball , R. Bhandari , G. M. Torres , et al., “CDDO‐Me Alters the Tumor Microenvironment in Estrogen Receptor Negative Breast Cancer,” Scientific Reports 10, no. 1 (2020): 6560. 32300202 10.1038/s41598-020-63482-x PMC7162855

[19] D. C. Goldman , V. Alexeev , E. Lash , C. Guha , U. Rodeck , and W. H. Fleming , “The Triterpenoid RTA 408 Is a Robust Mitigator of Hematopoietic Acute Radiation Syndrome in Mice,” Radiation Research 183, no. 3 (2015): 338–344. 25738896 10.1667/RR13900.1 PMC5826655

[20] Y. Y. Wang , H. Zhe , and R. Zhao , “Preclinical Evidences Toward the Use of Triterpenoid CDDO‐Me for Solid Cancer Prevention and Treatment,” Molecular Cancer 13 (2014): 30. 24552536 10.1186/1476-4598-13-30 PMC3940295

[21] X. Sun , Z. Xie , B. Hu , et al., “The Nrf2 Activator RTA‐408 Attenuates Osteoclastogenesis by Inhibiting STING Dependent NF‐Kappab Signaling,” Redox Biology 28 (2020): 101309. 31487581 10.1016/j.redox.2019.101309 PMC6728880

[22] S. A. Reisman , D. A. Ferguson , C. I. Lee , and J. W. Proksch , “Omaveloxolone and TX63682 Are Hepatoprotective in the STAM Mouse Model of Nonalcoholic Steatohepatitis,” Journal of Biochemical and Molecular Toxicology 34, no. 9 (2020): e22526. 32410268 10.1002/jbt.22526 PMC9285621

[23] P. S. Rabbani , T. Ellison , B. Waqas , et al., “Targeted Nrf2 Activation Therapy With RTA 408 Enhances Regenerative Capacity of Diabetic Wounds,” Diabetes Research and Clinical Practice 139 (2018): 11–23. 29476889 10.1016/j.diabres.2018.02.021

[24] R. Abeti , A. Baccaro , N. Esteras , and P. Giunti , “Novel Nrf2‐Inducer Prevents Mitochondrial Defects and Oxidative Stress in Friedreich's Ataxia Models,” Frontiers in Cellular Neuroscience 12 (2018): 188. 30065630 10.3389/fncel.2018.00188 PMC6056642

[25] J. Sun , J. Y. Li , L. Q. Zhang , et al., “Nrf2 Activation Attenuates Chronic Constriction Injury‐Induced Neuropathic Pain via Induction of PGC‐1alpha‐Mediated Mitochondrial Biogenesis in the Spinal Cord,” Oxidative Medicine and Cellular Longevity 2021 (2021): 9577874. 34721761 10.1155/2021/9577874 PMC8554522

[26] W. Jian , H. Ma , D. Wang , et al., “Omaveloxolone Attenuates the Sepsis‐Induced Cardiomyopathy via Activating the Nuclear Factor Erythroid 2‐Related Factor 2,” International Immunopharmacology 111 (2022): 109067. 35908503 10.1016/j.intimp.2022.109067

[27] W. Winardi , Y. P. Lo , H. P. Tsai , Y. H. Huang , T. T. Tseng , and C. L. Chung , “CDDO, an Anti‐Inflammatory and Antioxidant Compound, Attenuates Vasospasm and Neuronal Cell Apoptosis in Rats Subjected to Experimental Subarachnoid Hemorrhage,” Current Issues in Molecular Biology 46, no. 5 (2024): 4688–4700. 38785551 10.3390/cimb46050283 PMC11119475

[28] R. Zhou , Z. Liu , T. Wu , et al., “Machine Learning‐Aided Discovery of T790M‐Mutant EGFR Inhibitor CDDO‐Me Effectively Suppresses Non‐Small Cell Lung Cancer Growth,” Cell Communication and Signaling: CCS 22, no. 1 (2024): 585. 39639305 10.1186/s12964-024-01954-7 PMC11619116

[29] G. Luo , K. Aldridge , T. Chen , et al., “The Synthetic Oleanane Triterpenoid CDDO‐2P‐Im Binds GRP78/BiP to Induce Unfolded Protein Response‐Mediated Apoptosis in Myeloma,” Molecular Oncology 17, no. 12 (2023): 2526–2545. 37149844 10.1002/1878-0261.13447 PMC10701780

[30] L. J. Rogers , T. John , J. Park , et al., “Growth Inhibition and Apoptosis of Human Multiple Myeloma Cells Induced by 2‐Cyano‐3,12‐Dioxooleana‐1,9‐Dien‐28‐Oic Acid Derivatives,” Anti‐Cancer Drugs 31, no. 8 (2020): 806–818. 32304407 10.1097/CAD.0000000000000941

[31] S. B. Kim , R. G. Bozeman , A. Kaisani , et al., “Radiation Promotes Colorectal Cancer Initiation and Progression by Inducing Senescence‐Associated Inflammatory Responses,” Oncogene 35, no. 26 (2016): 3365–3375. 26477319 10.1038/onc.2015.395 PMC4837107

[32] J. Hao , J. Zhou , S. Hu , et al., “RTA 408 Ameliorates Diabetic Cardiomyopathy by Activating Nrf2 to Regulate Mitochondrial Fission and Fusion and Inhibiting NF‐kappaB‐Mediated Inflammation,” American Journal of Physiology. Cell Physiology 326, no. 2 (2024): C331–C347. 38047307 10.1152/ajpcell.00467.2023

[33] B. L. Probst , I. Trevino , L. McCauley , et al., “RTA 408, A Novel Synthetic Triterpenoid With Broad Anticancer and Anti‐Inflammatory Activity,” PLoS One 10, no. 4 (2015): e0122942. 25897966 10.1371/journal.pone.0122942 PMC4405374

[34] S. Y. Tam and H. K. Law , “JNK in Tumor Microenvironment: Present Findings and Challenges in Clinical Translation,” Cancers 13, no. 9 (2021): 2196. 34063627 10.3390/cancers13092196 PMC8124407

[35] L. J. W. Pua , C. W. Mai , F. F. Chung , et al., “Functional Roles of JNK and p38 MAPK Signaling in Nasopharyngeal Carcinoma,” International Journal of Molecular Sciences 23, no. 3 (2022): 1108. 35163030 10.3390/ijms23031108 PMC8834850

[36] S. S. K. Yalamarty , N. Filipczak , X. Li , et al., “Mechanisms of Resistance and Current Treatment Options for Glioblastoma Multiforme (GBM),” Cancers 15, no. 7 (2023): 2116. 37046777 10.3390/cancers15072116 PMC10093719

[37] V. N. Ivanov , J. Wu , and T. K. Hei , “Regulation of Human Glioblastoma Cell Death by Combined Treatment of Cannabidiol, Gamma‐Radiation and Small Molecule Inhibitors of Cell Signaling Pathways,” Oncotarget 8, no. 43 (2017): 74068–74095. 29088769 10.18632/oncotarget.18240 PMC5650324

[38] M. Okada , A. Sato , K. Shibuya , et al., “JNK Contributes to Temozolomide Resistance of Stem‐Like Glioblastoma Cells via Regulation of MGMT Expression,” International Journal of Oncology 44, no. 2 (2014): 591–599. 24316756 10.3892/ijo.2013.2209

[39] A. Zeng , J. Yin , Y. Li , et al., “miR‐129‐5p Targets Wnt5a to Block PKC/ERK/NF‐kappaB and JNK Pathways in Glioblastoma,” Cell Death & Disease 9, no. 3 (2018): 394. 29531296 10.1038/s41419-018-0343-1 PMC5847604

